# ALK阳性非小细胞肺癌患者克唑替尼耐药的机制和治疗措施

**DOI:** 10.3779/j.issn.1009-3419.2015.02.03

**Published:** 2015-02-20

**Authors:** 涛 蒋, 彩存 周

**Affiliations:** 200433 上海，上海市肺科医院肿瘤科 Department of Medical Oncology, Shanghai Pulmonary Hospital, Shanghai 200433, China

**Keywords:** 肺肿瘤, 克唑替尼, 间变性淋巴瘤激酶, 耐药性, Lung neoplasms, Crizotinib, Anaplastic lymphoma kinase, Drug resistance

## Abstract

肺癌是全球发病率和致死率最高的疾病之一。非小细胞肺癌（non-small cell lung cancer, NSCLC）是肺癌最为常见的组织学类型。近些年，分子生物学的发展让我们对NSCLC的认识从组织水平深入到分子水平。表皮生长因子受体（epidermal growth factor receptor, *EGFR*）基因突变和间变性淋巴瘤激酶（anaplastic lymphoma kinase, *ALK*）融合基因是NSCLC患者最为重要的两个肿瘤驱动基因。针对它们的酪氨酸激酶抑制剂（tyrosine kinase inhibitors, TKIs）显著改善了带有这类分子特征的NSCLC患者的生存。不幸的是，目前几乎所有针对这两种突变的初始靶向治疗都会不可避免地出现耐药问题。有关EGFR-TKIs的耐药机制及其应对策略已经有很多文章进行阐述，而对于ALK TKIs治疗后出现耐药问题的机制和相应的治疗策略还未曾有过详细的综述。因此，本文针对一代ALK TKI（克唑替尼）治疗ALK融合基因阳性的NSCLC患者（ALK+ NSCLC）后引起耐药问题的机制和有关后续治疗策略做一综述。

## Abstract (en)

Lung cancer is the common lethal disease with the highest morbidity and mortality worldwide. Non-small cell lung cancer (NSCLC) is the most common histological type of lung cancer. Recently, the advances in the molecular biology have transformed our view of NSCLC from histopathological descriptions to precise molecular and genetic identities that can be resolved to single-cell level. Epidermal growth factor receptor (*EGFR*) and anaplastic lymphoma kinase (*ALK*) are the most crucial tumor driver genes in NSCLC. Their tyrosine kinase inhibitors (TKIs) can significantly improve survival of these patients, who have these driver genes' mutation. Unfortunately, drug resistance would inevitably occur after almost all of initial targeted therapy. The resistance mechanism and corresponding methods on EGFR-TKIs have been reviewed elsewhere and will not be discussed. We will focus on the mechanism of first generation ALK TKI (crizotinib) resistance in patients with ALK positive NSCLC. Likewise, we will also discuss the current therapies for ALK positive NSCLC patients after crizotinib resistance.

肺癌是全球发病率和致死率最高的疾病之一。2014年世界卫生组织发布《World Cancer Report 2014》。该报告显示，肺癌以每年180万的新发病例和160万的死亡病例，高居所有癌症发病率和死亡率之首。报告同时指出，在我国，随着空气污染等问题的日趋严重，每年肺癌新发病例为67.6万，死亡病例56.5万，均居世界各国肺癌发病率和死亡率的第一位。非小细胞肺癌（non-small cell lung cancer, NSCLC）是肺癌最为常见的组织学类型，约占总体的80%-85%^[1-3]^。近些年，随着分子生物学和转化医学的快速发展，人们对NSCLC的认识已经从组织水平深入到分子水平，越来越多的肿瘤驱动基因被陆续鉴定出来。而针对这些驱动基因的靶向药物已经取代传统的化疗，成为带有这些驱动基因的晚期NSCLC的标准治疗方案。在NSCLC中最具有代表性的靶向药物是表皮生长因子受体（epidermal growth factor receptor, EGFR）和间变性淋巴瘤激酶（anaplastic lymphoma kinase, ALK）酪氨酸激酶抑制剂（tyrosine kinase inhibitors, TKIs）。相关的Ⅲ期临床试验也证实了这些TKIs可以使带有*EGFR*突变和（或）*ALK*融合基因的NSCLC患者得到明显的缓解和生存获益^[4-6]^。不幸的是，目前几乎所有的TKIs在初始治疗后的9个月-12个月都会出现耐药问题^[7, 8]^。耐药现象已经成为TKIs临床应用最普遍也是最大的障碍，同时也是提高晚期NSCLC患者生存时间最亟待解决的问题。有关EGFR-TKIs的耐药机制及其应对策略已经有很多文章进行阐述^[9-12]^，而对于ALK TKIs治疗后出现耐药问题的机制和相应的治疗策略还未曾有过详细的综述。因此，本文针对一代ALK TKI（克唑替尼）治疗*ALK*融合基因阳性的NSCLC患者（ALK+ NSCLC）后，引起耐药问题的机制和有关后续治疗策略做一综述。

## 1 克唑替尼治疗后的耐药机制

克唑替尼是一种口服型小分子ATP竞争性的ALK抑制剂，它可以抑制c-Met激酶，破坏c-Met的信号转导通路，进而抑制*ALK*融合基因，达到抑制肿瘤细胞生长的效果^[13, 14]^。*ALK*融合基因发生于3%-7%的NSCLC患者^[15, 16]^，临床上常见于不吸烟的年轻腺癌患者，通常与*EGFR*或*KRAS*突变的发生互相排斥^[17]^。克唑替尼的出现显著改善了该类亚型NSCLC患者的预后，其治疗客观缓解率（objective response rate, ORR）达60%，无进展生存期（progression free survival, PFS）达8个月-10个月，总生存期（overall survival, OS）显著延长^[18-20]^。尽管ALK+ NSCLC患者的获益明显，但这部分患者往往在1年-2年内出现对克唑替尼耐药，且中枢神经系统的复发进展较为常见^[21]^。已经发现的耐药机制主要有以下几种。

### 1.1 ALK继发性耐药突变

ALK+ NSCLC患者出现继发性耐药问题，约37%归因于ALK继发性耐药突变。这其中又可以分为ALK激酶区突变（28%）和*ALK*基因拷贝数扩增（9%）。ALK激酶区突变是最早明确的耐药机制^[22, 23]^。体外和基于患者标本的研究揭示ALK+ NSCLC继发耐药的患者存在数种ALK激酶区突变，包括：L1196M、L1152R、G1202R、G1269A、1151Tins、S1206Y、C1156Y、F1174C和D1203N。数量上微占优势的是L1196M，它是一种类似于T790M的看家基因^[24, 25]^。在首例公开报道的克唑替尼耐药的病例的同一个肿瘤标本中即发现了L1196M和C1156Y的突变。Doebele等^[22]^对14例经克唑替尼治疗后出现继发性耐药的ALK+ NSCLC患者的标本进行荧光原位杂交（fluorescence *in situ* hybridization, FISH）检测，结果发现三分之一的患者出现了ALK激酶区的二次突变，主要为L1196M、G1269A和C1156Y。与之相似，Katayama等^[23]^对18例克唑替尼获得性耐药的ALK+ NSCLC患者进行了FISH和免疫组织化学法（immunohistochemistry, IHC）检测，发现约四分之一的患者出现了ALK激酶区的二次突变，并且在体外实验中证实了这些突变导致了ALK+肿瘤细胞对克唑替尼的耐药。*ALK*融合基因拷贝数扩增首次是在ALK+细胞系出现对克唑替尼耐药时发现的，随后在对克唑替尼耐药的ALK+ NSCLC患者标本中也发现了拷贝数增加。在Katayama等^[23]^的试验中，FISH检测18例继发性耐药的患者的标本也发现了1例患者*ALK*融合基因拷贝数扩增。同样，相应的体外实验也证实了*ALK*融合基因拷贝数扩增可以导致ALK+肿瘤细胞对克唑替尼的耐药。值得一提的是，当*ALK*融合基因激酶区突变或拷贝数扩增时，ALK信号通路往往会被保留，并在肿瘤的生存和耐药过程中发挥一定的作用。因此，更加有效的第二代ALK抑制剂也许能够克服这些机制引起的继发性耐药问题。该类型的耐药也被称为ALK占优势的耐药。

### 1.2 驱动基因转换

ALK+肿瘤细胞主要通过ALK及其下游信号通路来控制肿瘤细胞的生长和迁移。当使用克唑替尼阻断该信号通路时，肿瘤细胞会通过另外一些机制（如转换驱动基因）来激活其他信号通路，取代肿瘤细胞对ALK及其下游信号的依赖，导致克唑替尼不能有效地抑制肿瘤细胞的生长。该类型的耐药又被称为ALK不占优势的耐药。这些转换驱动基因中最为常见的是*EGFR*突变或磷酸化、*KRAS*突变和*c-KIT*扩增。Doebele等^[22]^和Katayama等^[23]^的研究均发现，在未接受和已经接受克唑替尼治疗的ALK+ NSCLC患者的标本中均发现激活的*EGFR*或*KRAS*的突变。Katayama等^[23]^对18例克唑替尼耐药的标本进行IHC检测，发现有17例标本出现EGFR磷酸化，提示存在不同程度的EGFR通路激活。在细胞系的研究中发现，抑制EGFR磷酸化可以恢复耐药细胞株对克唑替尼的敏感性。另外，上述研究中，18例标本经FISH检测发现了2例高水平的c-KIT扩增，IHC检测也证实了这一结果。同时通过IHC检测还发现在这些c-KIT扩增的耐药标本的间质细胞中，c-KIT的配体干细胞因子（stem cell factor, SCF）也存在高表达现象。体外实验证实了c-KIT扩增介导的克唑替尼耐药现象需要高表达的SCF的参与，而克唑替尼联合伊马替尼则可以有效抑制这种机制导致的耐药现象。

### 1.3 肿瘤异质性

NSCLC是基因和细胞异质性最大的肿瘤之一。尽管最近几十年分子生物学技术的发展，让我们对NSCLC的认识从组织病理的水平深入到分子和遗传甚至单细胞水平，但是，对于肿瘤异质性及其导致有关的耐药问题，我们仍然缺乏更深入的了解^[8]^。事实上，我们已经在多种耐药肿瘤中观察到肿瘤异质性的存在。比如，对同一个ALK+ NSCLC患者的耐药肿瘤标本进行检测，分别鉴定出了两种不同的激酶区突变，而一些被鉴定的肿瘤细胞不存在这些突变。另一个患者的耐药标本则同时检测出了基因拷贝数扩增和突变^[26, 27]^。但是目前并不知道这些变异是否都存在于相同的细胞内。2014年美国临床肿瘤学会（American Society of Clinical Oncology, ASCO）会议上报道了一个关于多发肺结节的NSCLC患者驱动基因的分布情况。研究纳入35例双肺多发肺结节的NSCLC患者，分别检测其驱动基因分布情况，结果发现不同结节的突变驱动基因的不一致率达到68.6%。这些结果都表明：对于NSCLC患者而言，在不同时间和不同空间其肿瘤细胞可能存在不同的驱动基因突变。那么，一块很小的活检组织的分子检测结果能否代表整个肿瘤组织？有限的检测能否显示所有的细胞耐药的类型？肿瘤细胞异质性的存在让耐药问题更加错综复杂，要彻底解决耐药问题，首先需要更多地了解肿瘤异质性的起源等问题。

## 2 克唑替尼耐药后的治疗策略

### 2.1 二代ALK抑制剂

第二代ALK抑制剂的结构和克唑替尼有很大的不同，因而能够抑制ALK继发性耐药突变。临床前研究也已经证实，第二代ALK抑制剂不仅对存在ALK融合基因阳性的肿瘤细胞具有活性，而且对已经鉴定出来的多种ALK激酶区耐药突变均具有活性。这些药物包括：alectinib（CH5424802）、ceritinib（LDK-378）和AP26113。

#### 2.1.1 Alectinib

Alectinib是一种强效的选择性ALK抑制剂，其效力比克唑替尼强10倍，能有效对抗大多数的ALK激酶区突变^[28]^。在日本进行的一项多中心、单臂开放的Ⅰ期/Ⅱ期研究对未接受克唑替尼治疗的晚期NSCLC患者进行的剂量递增试验，试验中患者每日口服alectinib两次^[29, 30]^。Ⅰ期试验纳入的24例患者每日口服20 mg-300 mg的alectinib两次。在最高最大剂量耐受组未观察到剂量限制性毒性，也未见4级不良事件的报告。因此Ⅱ期临床研究选取了每次300 mg、每日两次的用药方案。共计46例患者以推荐剂量进行了治疗，其中43例患者获得客观缓解，客观缓解率（objective response rate, ORR）达93.5%（95%CI: 82%-98.6%），其中2例（4.3%）为完全缓解。12例（26%）患者出现了治疗相关的3级不良反应，包括中性粒细胞减少和血液肌酸激酶含量升高。5例患者出现了严重的副作用（11%）。中位PFS尚未达到。基于这一结果，日本卫生部、劳动和福利局批准了该药的使用。同样在美国，食品与药物管理局（Food and Drug Administration, FDA）已经批准alectinib用于克唑替尼治疗后发生进展的ALK+ NSCLC患者的治疗，并授予该药突破性治疗的称号。

#### 2.1.2 Ceritinib

Ceritinib是瑞士诺华公司研发的二代ALK抑制剂。其体外研究^[31]^表明：ceitinib对ALK+并表达突变C1156Y的肿瘤细胞均具有良好的活性。Ceritinib的剂量递增Ⅰ期单臂试验计划入组131例ALK+的经过标准治疗失败的进展期肿瘤患者^[32]^。试验分为3个组; 之前接受过克唑替尼治疗的ALK+ NSCLC，之前未接受过克唑替尼治疗的ALK+ NSCLC和ALK+的除肺癌之外的其他恶性肿瘤。截止2012年11月8日，共入组130例患者，其中59例为剂量爬坡组，确认最大耐受剂量（maximum tolerated dose, MTD）为750 mg/d; 后续71例患者为MTD扩展组。在79例对克唑替尼耐药的ALK+ NSCLC亚组中，ORR为57%。剩余35例未经克唑替尼治疗的ALK+ NSCLC组中，ORR为60%。在114例NSCLC患者中，中位PFS为8.6个月（95%CI: 5.7-9.9）。最常见的不良反应为恶心（73%）、腹泻（72%）、呕吐（58%）和疲劳（41%）。最常见的3级-4级不良事件为丙氨酸氨基转移酶升高（19%），天冬氨酸氨基转移酶升高（10%）和腹泻（8%）。该项研究结果表明LDK378在400 mg/d-750 mg/d剂量范围内显示出较强的抗肿瘤活性（无论其是否接受过克唑替尼治疗）。最常见的不良反应为恶心、腹泻、呕吐和乏力，且多为1级或2级，患者耐受性较好。目前ceritinib已经被FDA批准用于进行多项其他Ⅱ期和Ⅲ期的临床研究。

#### 2.1.3 AP26113

AP26113是一种新型的ALK-/EGFR-TKI双重抑制剂，可强效抑制*ALK* L1196M突变和*EGFR* T790M突变^[33, 34]^。一项开放标签、多中心Ⅰ期/Ⅱ期临床试验共纳入44例晚期实体瘤病例，37例是耐受现有治疗药物或没有标准治疗的NSCLC。患者每日口服1次的AP26113。与治疗相关的3级-4级最常见不良事件为腹泻（5%）。两种剂量限制性毒性出现在240 mg剂量组（丙氨酸转氨酶升高3级事件）和300 mg剂量组（呼吸困难4级事件）。Ⅱ期临床试验以 < 300 mg的剂量进一步研究，设4个受试群体：ALK+ NSCLC初治组、ALK靶向治疗后耐药组、耐药*EGFR*突变型组、其他ALK变异或其他以AP26113为靶点的肿瘤。

综上所述，第二代ALK抑制剂对于仍依赖于ALK信号通路的ALK激酶区耐药突变也许是最佳的选择。

### 2.2 联合其他治疗

上文提到，不占优势的ALK耐药突变存在其他信号旁路激活的现象。因此，应用ALK抑制剂和其他信号传导通路抑制剂，有可能改善此类机制导致耐药问题的临床疗效。

#### 2.2.1 联合热休克蛋白（heat shock protein 90, HSP90）抑制剂

HSP90是一类称为“分子伴侣”的蛋白质，可帮助新合成的蛋白质形成发挥他们特定生物学功能的正确形状^[35]^。体外细胞系研究中发现HSP90的抑制剂ganetespib对ALK+的未经过克唑替尼处理或对克唑替尼耐药的细胞系均有活性^[21, 36]^。一项关于ganetespib的Ⅱ期临床试验中^[37]^，ganetespib单药治疗*ALK*基因重排的IIIb期/Ⅳ期NSCLC，患者之前未接受过直接ALK抑制剂治疗。主要终点是ORR，计划招募受试者约100例。目前试验正在进行中，已经报道的结果显示4例获部分缓解的病例均为未经克唑替尼治疗的ALK阳性型。最常见不良反应为腹泻、乏力、恶心和食欲减退。另一种HSP90抑制剂是AUY922^[38]^，目前正在进行用于包括ALK+ NSCLC在内的Ⅱ期试验。剂量为每周70 mg/m^2^，121例受试者均为复治NSCLC患者。22例ALK+病例中，客观缓解7例（32%），其中3例是克唑替尼耐药者; 疾病控制率为59%（未经克唑替治疗组为100%，克唑替尼耐药组为36%）。不良事件主要是1级-2级事件，包括眼睛不适（77%）、腹泻（74%）和恶心（46%）。其他联合HSP90抑制剂与选择性ALK抑制剂的临床研究正在进行中（NCT 01712217和NCT01579994）。

#### 2.2.2 联合其它TKI

既往的研究^[39, 40]^表明，在ALK+ NSCLC患者中，5%-8%的ALK+细胞并存*EGFR*突变。在基于亚裔患者的研究^[27]^中发现，18.6% ALK+患者合并*EGFR*突变; 3.9%的*EGFR*突变患者合并ALK融合。对于这些患者，联合使用EGFR和ALK TKI相对于单独使用任何一种TKI可以起到更好的抑制效果。但是目前该结论多见于病例报道研究，尚缺乏相关的大型临床数据的支持。

#### 2.2.3 联合化疗

*EGFR*突变阳性NSCLC患者对EGFR-TKI耐药后，立即停止EGFR-TKI治疗可导致肿瘤出现“闪耀”现象（即暴发性进展），可能的原因是停止EGFR-TKI治疗后，对EGFR-TKI敏感的细胞再次快速增殖，导致肿瘤暴发性生长。同理，对克唑替尼敏感的ALK+ NSCLC亦可出现相似的过程。因此，当该类患者出现对克唑替尼获得性耐药时，在全身化疗同时是否需要继续克唑替尼治疗，尚无确切答案，通过一些前瞻性临床研究有助于回答这一问题。正在设计的SWOG1300研究即是针对ALK+ NSCLC患者出现克唑替尼耐药后随机分配至培美曲塞单药或培美曲塞联合克唑替尼治疗组。值得关注的是，在这项研究中，如果单药培美曲塞治疗失败后，将允许患者再次接受克唑替尼治疗。基于此研究，亦能回答“在具有明确肿瘤驱动基因的患者出现获得性耐药后，两种不同的治疗模式孰优孰劣（继续原小分子靶向药物联合全身化疗*vs* 先全身化疗，待疾病再次进展后重新使用小分子靶向药物）”这一问题。研究结果将对临床实践具有重要的指导意义。

### 2.3 继续克唑替尼治疗

对于靶向药物治疗出现获得性耐药的NSCLC患者，一个很重要的观点是认为这种耐药往往不完全，因此当疾病进展时，仍有部分肿瘤细胞能够继续被靶向药物所抑制。一项关于继续使用克唑替尼抑制ALK+ NSCLC疾病进展的研究^[41]^发现，克唑替尼治疗ALK+ NSCLC患者疾病进展后，继续使用克唑替尼对比未使用克唑替尼，6个月总生存率为76.3% *vs* 31.2%，1年总生存率为64.7% *vs* 32.9%，OS为16.4个月*vs* 3.9个月。对其总生存的*Cox*生存分析发现，克唑替尼持续使用可以使生存获益（风险比：0.27，95%CI: 0.17-0.45，*P* < 0.000, 1）。而患者的性别、吸烟史、体力状态评分等对生存均无影响。对于具有驱动基因突变的NSCLC患者，在更改治疗方式之前，区分出仅可检测的疾病进展与临床进展非常重要。这是由于某些患者虽然出现了局部的、无症状性进展，但与接受靶向治疗之前相比，肿瘤负荷仍能有较好的控制。还有些患者发现疾病进展后停用TKI，出现了疾病暴发性进展。这时给予患者同一种TKI或作用于同一靶点的不同种TKI，疾病可再次获得良好控制。该现象说明TKI仍能抑制对其敏感的肿瘤细胞亚群。因此，区分不同的耐药模式对后续治疗至关重要。

## 3 结论

克唑替尼是继EGFR-TKI类药物用于治疗*EGFR*突变的NSCLC靶向药物后又一重要的小分子靶向抑制剂，其对带有*ALK*融合基因阳性的NSCLC患者显示了良好的临床效果。然而由于多种原因导致的耐药问题则限制了克唑替尼的临床应用。对于克唑替尼耐药机制的深入探讨将有助于我们改善这类患者的临床结局。我们应该清楚，目前出现的二代ALK抑制剂等药物，虽然可以克服一代ALK TKI的部分耐药问题，但是解决耐药问题的最佳方法还有待于我们对肿瘤异质性的深入研究。
